# Smoking: Bans, Bans, Good for the Heart!

**DOI:** 10.1289/ehp.114-a154b

**Published:** 2006-03

**Authors:** Adrian Burton

Could moving to Pueblo, Colorado, be a new way to reduce the risk of suffering a heart attack? A study reported at the American Heart Association’s Scientific Sessions 2005 Conference in Dallas last November suggests that the city’s ban on smoking in all public buildings helped reduce the local heart attack rate by 27% over a year and a half. The finding appears to bolster recent decisions to ban smoking in workplaces and other public buildings across the United States, Spain, Ireland, and parts of the United Kingdom.

Tobacco smoke—including secondhand smoke—activates blood coagulation pathways that could lead to coronary thrombosis, or heart attack. Smoke is particularly dangerous for people whose arteries are already hardened by age, cholesterol deposits, or smoking itself. Indeed, some 30% of heart attacks are thought directly related to smoke-induced thrombosis. In the United States, the annual direct medical cost of coronary heart disease caused by secondhand smoke alone is some $2.45 billion, according to the U.S. Society of Actuaries.

In an attempt to reduce the incidence of heart attacks and other smoking-related illnesses, Pueblo introduced a ban 1 July 2003 on smoking in all indoor public areas within the city limits. Eighteen months later the Pueblo Public Health Department instigated an observational study to determine whether the ban had had any effect on the incidence of coronary events.

“We recorded the number of heart attacks that occurred in the city for the eighteen months before and after the introduction of the ban,” explains Mori Krantz, director of the Prevention Department of the Colorado Prevention Center in Denver, “and found a post-introduction fall of around twenty-seven percent. This was significantly greater than the small reduction we saw among the population living outside the city limits, and much greater than the virtually unchanged rate among residents of [adjacent] El Paso County, which does not have such an ordinance.”

Similar findings were reported in an earlier study published 24 April 2004 in *BMJ* after a smoking ban was imposed in Helena, Montana. “Our study builds on this work by involving a sample three times as large, and suggests that these ordinances may be having a positive effect on cardiac health,” says Krantz.

“This observational study is limited in that it does not distinguish between smokers and nonsmokers, and did not check the medical records of those involved,” remarks Jose Ramón Banegas, a professor of preventive medicine and public health at the Universidad Autónoma in Madrid, Spain. He adds, however, that “even if the effect were half that reported, it would mean this type of ban could save many lives while reducing health spending.”

Banegas points out that the results are also relevant given the new Spanish ban, enacted 1 January 2006, which still allows restaurateurs to declare their locales either smoking or nonsmoking. “Unfortunately,” he says, “ninety-eight percent are scared of losing income by going smoke-free.”

